# Supplementary material for: Do conspiracy theories efficiently signal coalition membership? An experimental test using the “Who Said What?” design
Source: PLoS One. 2022 Mar 10;17(3):e0265211. doi: 10.1371/journal.pone.0265211 (PMC8912250; doi:10.1371/journal.pone.0265211)
Supplement: S1 File — (PDF) [file pone.0265211.s001.pdf]

Full list of statements

Below are the statements presented to participants in the conducted Who-Said-What experiments. Odd-number statements are pro-environmental whereas even-number ones are environmental-skeptic. Each statement has two versions, one where the justification for the given environmental position is framed in a non-conspiratorial way (NC) and one where it is framed in a conspiratorial way (C). Sentences in ordinary type are present in both conditions whereas those in italic vary across conditions.

To the extent possible, we sought to design similar justifications between the two conditions, which varied solely by the presence or absence of a conspiratorial dimension in order to maximize experimental control. To maximize ecological validity and minimize bias in the design of stimuli, we designed beliefs inspired from environmental statements available on the internet.

1.

NC - The government should do more to fight climate change. *It is happening fast.*

C - The government should do more to fight climate change. *The big banks deny that climate change is happening fast because they can make a profit off the victims of natural disasters.*

2.

NC - People should not worry about climate change. *Humans are not responsible for it.*

C - People should not worry about climate change. *All the scientists who claim that humans are responsible for climate change are lying to us.*

3.

NC - The Department of Agriculture should ban GMOs. *GMOs have a negative impact on the environment as well as on our health.*

C - The Department of Agriculture should ban GMOs. *For decades, agribusinesses have suppressed data showing that GMOs harm the environment and our health.*

4.

NC - The government should not do anything to protect biodiversity. *Polar bears are not disappearing from the planet.*

C - The government should not do anything to protect biodiversity. *Alarmist NGOs are making up stories about the disappearance of polar bears just to secure money for themselves.*

5.

NC - The government should take more measures to promote electric cars. *Local funding for building charging stations is too low.*

C - The government should take more measures to promote electric cars. *Oil companies made secret arrangements with local authorities to cut funding for building charging stations.*

6.

NC - The government should not listen to environmentalists about plant-based diets. *Eating meat is essential to get proteins.*

C - The government should not listen to environmentalists about plant-based diets. *It is a lie that you don't need to eat meat to get proteins and vegans have made it up to sell their own products.*

7.

NC - We need to find alternatives to capitalism fast. *Capitalism generates a huge amount of CO2.*

C - We need to find alternatives to capitalism fast. *Big companies are working hand in hand with politicians to hide from us how much CO2 their system generates.*

8.

NC - The government needs to stimulate economic growth. *Degrowth, as promoted by environmentalists, would harm our way of life.*

C - The government needs to stimulate economic growth. *The environmentalists promoting degrowth are being hired by foreign states to harm our way of life.*
