# Supplementary material for: Do conspiracy theories efficiently signal coalition membership? An experimental test using the “Who Said What?” design
Source: PLoS One. 2022 Mar 10;17(3):e0265211. doi: 10.1371/journal.pone.0265211 (PMC8912250; doi:10.1371/journal.pone.0265211)
Supplement: S2 File — (PDF) [file pone.0265211.s002.pdf]

Pre-test of statements

Following standard practice in experimental psychology[1,2], materials were pre-tested. 100 American participants (32 women; mean age = 36.9 years) were recruited on Amazon Turk in order to assess that the chosen statements met the study's criteria. Participants were compensated with pay.

Procedure

Participants had to rate all eight statements on four attributes using a seven-point scale (1 = strongly disagree, 7 = strongly agree). For each statement randomly displayed to be either the conspiratorial or non-conspiratorial version, participants had to report how much they agreed that the statement was: *1) pro-environmental, 2) conspiratorial, 3) a widely held belief, 4) offensive.*

Measures

Repeated-measures ANOVAs were performed to assess if there was:

- A significant difference in environmental position rating between "pro-environmental" statements and "environmental-skeptic" ones, both for the non-conspiratorial and conspiratorial versions of statements.
- A significant difference in conspiracy rating between non-conspiratorial and conspiratorial statements.
- A significant difference in the offensiveness rating between non-conspiratorial and conspiratorial statements.
- A significant difference in the "widely held belief" rating between non-conspiratorial and conspiratorial statements.

---

[1] Reips UD. Standards for Internet-based experimenting. Experimental psychology. 2002;49(4):243.
[2] Reips UD. The methodology of Internet-based experiments. The Oxford handbook of Internet psychology. 2007;373-390.

The first two criteria are methodologically essential as they assess the validity of the designed statements in terms of environmental position and conspiratorial dimension.

The last two criteria correspond to the *burning bridges* components on which the specified theory relies, namely that conspiracy theories act as efficient coalitional markers due to their fringe and offensive components. It is therefore important to ascertain that conspiratorial beliefs are indeed more fringe and offensive than non-conspiratorial ones.

In this work, we were interested mainly in a relative phenomenon, namely that beliefs possessing more "burning-bridges" components are more efficient in triggering categorization by environmental position than beliefs that are less prone to burning bridges. Thus, we chose the differences in ratings between statements as the relevant statistical test for our pre-test to be validated rather than absolute comparisons with the midpoint (3.5). Differences per statement were also controlled for in the analyses.

Results and conclusion

Statements designed to be "pro-environmental" were significantly rated as more pro-environmental ($M = 5.43$, $SD = 1.49$) than "environmental-skeptic" ones ($M = 3.48$, $SD = 2.16$); $F(1, 677.81) = 79.29$), $p < .001$. This result does not differ across conspiracy conditions as the interaction effect was not significant ($F(1, 728.87) = 1.92$, $p = .17$).

Statements designed to be "conspiratorial" were significantly rated as more conspiratorial ($M = 5.35$, $SD = 1.53$) than "non-conspiratorial" ones ($M = 4.36$, $SD = 1.96$); $F(1, 719.19) = 69.56$, $p < .001$.

Regarding *burning bridges* components, statements designed to be "conspiratorial" were significantly rated as more offensive ($M = 4.70$, $SD = 1.75$) than "non-conspiratorial" ones ($M = 4.06$, $SD = 1.98$); $F(1, 696.12) = 31.91$, $p < .001$. They were also less widely held ($M = 4.23$, $SD = 1.78$) than "non-conspiratorial" ones ($M = 4.51$, $SD = 1.70$); $F(1, 700.21) = 6.81$, $p = .009$.

The eight designed statements are therefore valid stimuli for our experiments.
