# Supplementary material for: Do conspiracy theories efficiently signal coalition membership? An experimental test using the “Who Said What?” design
Source: PLoS One. 2022 Mar 10;17(3):e0265211. doi: 10.1371/journal.pone.0265211 (PMC8912250; doi:10.1371/journal.pone.0265211)
Supplement: S3 File — (PDF) [file pone.0265211.s003.pdf]

Attention checks

Three attention checks were used to detect low attentiveness, low effort and trolling in the conducted studies.

a) Detection of low attentiveness

To detect participants displaying low attention, we used a “color test” running as follows: "*The colour test is simple, when asked for your favourite colour you must enter the word iris in the text box below.*

Based on the text you read above, what colour have you been asked to enter? (textbox)"

Participants who did not enter the word iris (or close variants such as "Iris" or typos such as "iriis") were excluded from the analyses.

b) Detection of low effort

Participants who did not invest any effort in the experiment were detected by looking at answers in the distractor task (listing as many countries as possible). Participants who did not write any country name were excluded from the analyses.

c) Detection of trolling

To detect trolling, we presented participants with the following set of questions: "*Are you deaf or have a hearing impairment? / Are you blind or have a vision impairment? / Are you in a gang? / Is one or more of your family members in a gang?*".

Participants who answered yes to all questions, an extremely low-frequency combination very likely to reflect trolling, were excluded from the analyses[1].

[1] Robinson-Cimpian, J. P. (2014). Inaccurate estimation of disparities due to mischievous responders: Several suggestions to assess conclusions. Educational Researcher, 43(4), 171-185.

Analyses without inattentive respondents

In the pre-registration of each study (Study 1 and Study 2 were preregistered on the Open Science Framework (OSF) at https://osf.io/6aumy and https://osf.io/43trw respectively), we planned to exclude participants who failed at least one of the attention checks described above. However, as the attention checks occured post-treatment, there can be a bias in estimated effects[2]. Therefore, results reported in the main body of the article are full-sample analyses. We here present the pre-registered analyses excluding inattentive respondents, yielding identical substantive conclusions.

Pilot study

When inattentive respondents are excluded from the analysis (leaving 108 participants: 42 women, mean age = 35.8 years), categorization scores remain significantly above zero for both race ($r = .40$, $p < .001$, 95% CI [0.24, 0.53]) and environmental position ($r = .25$, $p = .008$, 95% CI [0.06, 0.41]).

Study 1

When inattentive respondents are excluded from the analysis (leaving 785 participants: 434 women, mean age = 45.6 years), both in the control and the treatment condition participants categorized target speakers on the basis of environmental position (control: $r = .20$, $p < .001$, 95% CI [0.10, 0.29]; treatment: $r = .14$, $p = .004$, 95% CI [0.03, 0.23]) and race (control: $r = .47$, $p < .001$, 95% CI [0.39, 0.54]; treatment: $r = .37$, $p < .001$, 95% CI [0.28, 0.45]). These results replicate the findings of the pilot study, highlighting their robustness. As predicted, categorization by race was significantly lower in the treatment condition compared to the control condition ($t = 1.84$, $df = 734$, $p = .03$). Categorization by environmental position, however, was not significantly larger in the treatment condition ($t = 0.59$, $df = 732$, $p = .72$).

[2] Montgomery, J. M., Nyhan, B., & Torres, M. (2018). How conditioning on post treatment variables can ruin your experiment and what to do about it. *American Journal of Political Science*, *62*(3), 760-775.

Study 2

When inattentive respondents are excluded from the analysis (leaving 892 participants: 509 women, mean age = 46.3 years), both in the control and the treatment condition participants categorized target speakers on the basis of environmental position (control: $r = .19$, $p < .001$, 95% CI [0.10, 0.27]; treatment: $r = .26$, $p < .001$, 95% CI [0.17, 0.34]) and race (control: $r = .53$, $p < .001$, 95% CI [0.47, 0.59]; treatment: $r = .51$, $p < .001$, 95% CI [0.44, 0.57]). However, neither of our hypotheses were supported by the data. Despite a slight increase in categorization by environmental position, the change does not reach significance at conventional levels ($t = 1.21$, $df = 890$, $p = .11$). Neither was categorization by race significantly decreased in the treatment condition compared to the control condition ($t = 0.59$, $df = 890$, $p = .28$).
