# Supplementary material for: Do conspiracy theories efficiently signal coalition membership? An experimental test using the “Who Said What?” design
Source: PLoS One. 2022 Mar 10;17(3):e0265211. doi: 10.1371/journal.pone.0265211 (PMC8912250; doi:10.1371/journal.pone.0265211)
Supplement: Attachment — Submitted filename: Response to Reviewers (2).pdf [file pone.0265211.s005.pdf]

Dear Editor,

We are grateful for the opportunity to revise our manuscript "Do conspiracy theories efficiently signal coalition membership? An experimental test using the "*Who Said What?*" design" for PLOS ONE.

We wish to thank Reviewer 1 for assessing that the comments from the initial review were addressed and Reviewer 2 for writing that he/she "*applauds the authors for a well-executed revision. The study and its contributions are much more clear.*".

We implemented multiple modifications and clarifications to improve the manuscript following the recommendations of Reviewer 2. First, we replaced all the results reported in the main text by analyses performed on the full sample without exclusion of inattentive respondents. We therefore removed the appendix where those results were previously reported (S4) and instead added the pre-registered analyses with exclusion of inattentive respondents in the S3 File, after the description of the attention checks performed. As underlined by the reviewer, full-sample analyses should be reported in the main text to avoid the issue of post-treatment bias and in our case results remain unchanged, the only difference being a slight reduction in effect sizes. Figures 2 et 3 now depict these new results. We also clarified and nuanced the offensive dimension of conspiracy theories in the introduction and gave examples to clarify. Finally, we also modified the presentation of the design of Study 2 to clarify its aim and emphasize the differences with Study 1.

Please find below a detailed description of our revisions in response to the reviewers' comments. The original comments appear in *black italics*, whereas our responses appear in blue. We hope that you will find that the revised manuscript better fulfills the criteria for publication.

Best regards,
The authors

EDITOR

Please review your reference list to ensure that it is complete and correct. If you have cited papers that have been retracted, please include the rationale for doing so in the manuscript text, or remove these references and replace them with relevant current references. Any changes to the reference list should be mentioned in the rebuttal letter that accompanies your revised manuscript. If you need to cite a retracted article, indicate the article's retracted status in the References list and also include a citation and full reference for the retraction notice.

We have reviewed our reference list and ensured it is complete and correct. We have not cited papers which have been retracted. The only modification made to the reference list is its order (reference 39 became 33, so that references 33 to 38 became references 34 to 39).

REVIEWER 2

*1. If I were the authors, I would indeed report results for all subjects, including those who failed the post treatment attention check. The authors acknowledge that these results are what *should* be reported; the results don't change (they say) if those subjects are included; and, perhaps most importantly, this paper is going to be published, and it would be unfortunate if readers focused on this error, rather than the substantive contribution of the paper. In short, I think it's in their interest, and the long-term interests of this paper, to make this change.*

We have made the changes suggested by the reviewer, moving the results with exclusion of inattentive respondents in the supplementary information (in S3, after the description of attention checks) and replacing them in the main text with the results without exclusion (that were reported in S4 in the revision). We agree with the reviewer that it would be unfortunate if readers focused on the possible post-treatment bias when reading the article. Moreover, our conclusions remain unchanged, with only a slight reduction in effect sizes when inattentive respondents are included. We also modified the figures accordingly.

In the Participants section of all studies, we removed all references to attention checks and modified the number of participants included in the analyses. Also, when introducing Study 1, as we deviate from the pre-registration by not excluding inattentive respondents, we added the following paragraph:

> *"In our pre-registered studies, we planned to exclude participants who failed attention checks. However, because attention checks were implemented post-treatment, these exclusions could bias our causal estimates [33]. Accordingly, we deviate from our pre-registrations and include all respondents in the analyses reported below. In the supporting information (S3), we report pre-registered analyses on attentive respondents yielding identical substantive conclusions."* (p.11)

*2 I would appreciate more details on the modifications made in Study 2. Right now, I don't think I fully grasp how the alignment of "conspiratorial dimension of statements with environmental position in the treatment condition" resulted in "all conspiratorial statements [being] either pro-environmental or environmental-skeptic." I *think* what the authors are trying to say is that they wanted to evaluate categorization by conspiracism in general, not categorization by conspiracism by environmental position. They should clarify on this point (and offer examples.)*

We agree with the reviewer on the need to clarify the design of Study 2, its aim and its differences with Study 1. We realized that it may be clearer to describe Study 2 as an experiment eliminating a confound rather than testing an alternative hypothesis. Indeed, we believed that the unexpected results of Study 1 may be due to the fact that people categorize targets according to conspiracism in general, and thus that having only conspiratorial statements in our treatment condition may be a confounding factor blurring categorization by environmental position. We therefore wished to make conspiracism vary in our new design, which is why only half of the statements in the treatment condition are conspiratorial in Study 2. Because we were still mainly focusing on the potential use of conspiratorial sentences to strengthen categorization across another coalitional dimension, we did not create a new conspiracy dimension orthogonal to race and environment. Instead, we aligned the conspiratorial dimension with environmental position such that either all four pro-environmental statements are conspiratorial and no environmental-skeptic statements are, or vice versa. We then tested whether conspiratorial arguments strengthen categorization by environmental position if only one side uses them. We therefore reframed both the discussion of Study 1, the introduction of Study 2 and its conclusion to clarify these points:

> *"(...) A possible **confound** influencing the results of this study is that conspiratorial justifications could serve as an indicator of affiliation with an independent coalition composed of all conspiracy theorists (...) " (p. 13, discussion of Study 1)*

> *"Study 2 was designed to investigate further the unexpected results of Study 1, **by eliminating the potential confound that conspiratorial justifications may serve as an indicator of affiliation with an independent coalition composed of all conspiracy theorists. To do so, we modify the treatment condition by eliminating half of the conspiratorial frames compared to Study 1. As our focus remains on the potential use of conspiratorial sentences to boost categorization across another coalitional dimension, we do not create a new conspiracy dimension orthogonal to race and environment. Instead, we align the conspiratorial dimension with environmental position such that either all four pro-environmental statements are conspiratorial and no environmental-skeptic statements are, or vice versa. We then test whether conspiratorial arguments strengthen categorization by environmental position if only one side uses them. If this is true, we expect categorization by environmental position to increase in the treatment group compared to the control group, as all conspiracy theorists now share the same environmental stance. Furthermore, if indeed conspiratorial asymmetries boost environmental position as a coalitional cue, we expect categorization by race to decrease in the treatment group.**"* (p.14-15)

> *"(...) Hence, the findings of Study 2 do not support the prediction that **conspiratorial frames boost categorization by environmental position when only one side uses them**, as only a weak effect in the expected direction was found."* (p.16)

Finally, we modified the general discussion to clarify the findings of Study 2:

*"Study 2 was designed **to eliminate a confound that could influence Study 1's results, namely that conspiratorial justifications may serve as an indicator of***

affiliation with an independent coalition composed of all conspiracy theorists. However, Study 2 only found a weak effect in favor of the coalitional cue conveyed by conspiracy theories when removing this confound.*”* (p. 16-17)

3. Finally, I admit I don't fully understand why conspiracy theories are inherently "offensive." Consider those who believe in JFK assassination theories. Given how widely held such beliefs are among U.S. citizens, it's hard to understand how the belief itself is "offensive" in any meaningful way. The authors should either explain this term or use a more precise one.

We agree with the reviewer that conspiracy theories can take multiple forms and thus sometimes do not appear as explicitly offensive. In the case of JFK assassination conspiracy theories, some groups were accused of the assassination such as the CIA, the Mafia, Lyndon Johnson, Fidel Castro, the KGB, etc. But it is true that sometimes the theory just runs as “JFK was assassinated” and that the reference to malevolent groups is rather implicit. We have reframed and nuanced the part of the introduction where the offensive dimension of conspiracy theories is discussed to reflect more diverse forms of conspiracy theories (including examples):

> *“A conspiracy theory is commonly defined as the belief that a group of agents secretly acts together with malevolent intent [18,19].* ***Most conspiracy theories are thus inherently offensive: they accuse some actors of harming innocent people, either actively (as in the chemtrail conspiracy) or passively by concealing relevant information and “covering up tracks”. Another common case is that conspiracy theories deny grievances or important achievements of certain actors (e.g. Holocaust deniers or the 9/11 Truth Movement; moon-landing hoax), thereby also fostering inter-group conflict.*** *Moreover, many conspiracy beliefs oppose mainstream narratives and are often held by small minorities* ***(e.g. Reptilian conspiracies)****, thereby also possessing a fringe element.” (p.4)*

But again, this is a strong revision. I look forward to reading the published version.
Thank you!
